# Modern Sociology

**Published:** 1896-11-14

**Authors:** 


### Modern Sociology.

### MISTAKEN IMPRESSIONS OF PRISON LIFE.—II.

In the long list of misconceptions as to the interior arrangements of prisons, none is more firmly fixed in the popular mind than the certainty of contaminating influences resulting from the association of greater and lesser offenders together. Prisons seem to be looked upon by that vast number of persons who have no opportunity of really informing themselves on the subject as a species of university for the higher education in crime, where young criminals are carefully coached in nefarious practices by their older associates. Now, there is simply no such association in the true sense of the word. We do not answer for what may have been the case in former times, but we are speaking now from a personal experience of over twenty years, and we affirm positively that, according to present regulations, no intercourse is possible between prisoners which could produce a corrupting effect. We make this statement of English prisons only; we are aware that it is otherwise in some foreign countries. We have ourselves seen in a large French gaol an indiscriminate assemblage of most ruffianly-looking convicts who had every facility for conversing together as much as they pleased. In England, however, as well as in Scotland and Ireland, such evils are guarded against very rigidly. The officials in authority in these days belong to a much higher class than those employed in that way at an earlier date. They are now for the most part cultivated and right-minded men and women, who understand the reasons of the disciplinary rules they have to enforce, and take care that they shall be carried out, so as to ensure the definite fulfilment of their purpose. To prevent the smallest intercourse of any kind between the prisoners is one of their paramount duties, and it is well and successfully performed. Each prisoner has, of course, his separate cell, to which he is rigidly confined, excepting when engaged in any species of labour that is shared by other inmates. When employed in such duties they are placed at a certain distance apart, and watched in every movement by the warders in attendance, so that communication between them is quite impossible. It is generally known, we believe, that when they are ordered to exercise they follow each other in single file round an encircling path, seeing only the backs of their comrades, with a considerable space marked out between each individual, while officers in the centre of the circle keep an unrelenting watch upon them, and pounce on any attempt to communicate with each other, even only by signs. We fully admit that the prisoners themselves make every conceivable effort to hold intercourse of some kind with their fellow culprits, if only to relieve the silence and solitude—intolerable to persons of their class, who have not sufficient cultivation of mind to supply them with food for thought—and though they are very ingenious in their expedients to that end, their most successful attempts could never amount to such a degree of correspondence with each other as would render instruction in crime possible. Knocking on the walls of separation between the cells, scratching sentences on the sides of the baths or the bottom of the tins used to contain their gruel, and many other devices of that inadequate nature, are instantly detected and stopped by the officials. The chapel is perhaps the most favourable ground for enabling them to let their presence at least be known to acquaintances who have been incarcerated at an earlier or later period from themselves. The male and female prisoners are, of course, rigidly separated during the services. A high and strong wooden partition divides the portion of the building they respectively occupy, but they do not allow this serious obstacle to deter them altogether from the communications they specially desire to hold with the opposite sex. In singing the hymns they often try to introduce words of their own, or make very peculiar responses, which can be understood over the wall. A male prisoner will be afflicted with an extremely bad cough, which, in measured attacks, makes known to a lady friend on the other side that he is "in quod," but he is seldom oppressed by this bronchial malady on more than one occasion, since the governor informs him that, as his cough is so distressing, he is to remain in his cell and not be exposed to the air of the chapel until he is better, a cure for his complaint which is at once perfectly complete. On the female side of the partition a woman permitted to take her infant, born in prison, to chapel with her, pinches the unfortunate mite till its shrill yells reveal her proximity to its father attentively listening through the wall.

Recently the governor of one of our county prisons was greatly perplexed by the discovery that the female criminals in his charge managed in some mysterious manner to ascertain the presence of every individual man on the other side of the impervious dividing barrier. One of the women inadvertently let drop the fact that she had recognised her husband, whose position there must, according to rule, have been completely unknown to her. None of the officers could account for an unpermitted knowledge which was found to be shared by all the other women. At last a very careful examination of the chapel gave an explanation of the mystery. Although strictly divided, as we have said, both the male and female prisoners faced the altar in their seats, and over it had been fixed a very large brass cross against the wall, so highly polished as to form a very good mirror. In its clear surface the women saw the reflection of every man as he passed to his place, and had enjoyed the spectacle with impunity, till a wife, much interested in the appearance of her spouse, had made an imprudent remark to one of the officers, which revealed the fact. The brass cross instantaneously disappeared, and the blank wall behind it no longer tells any secrets. The details of these tricks might be greatly multiplied, but we have said enough to show that the utmost ingenuity of prisoners could never compass sufficient opportunity for the smallest attempt at the use of a corrupting influence. The idea of this risk has been partly the cause, for a considerable time, of magisterial action with regard to boys convicted of penal offences. They are almost invariably sent to reformatories in order to avoid committing them to prison. It is, unfortunately, a fact, known to all persons cognisant of these matters, that the youngsters are infinitely more exposed to contamination in these schools, where of course they mix more or less with their companions, than they could possibly be in the carefully watched and rigorously guarded prison. The care of these immature little rogues has, however, resulted in a most salutary measure by the creation of the "First Offenders" Act, with which in its detail and results we shall hope to deal at our next opportunity.

